# Degradation of BiTeCl induced by thermal and laser treatment

**DOI:** 10.1038/s41598-025-00907-5

**Published:** 2025-05-07

**Authors:** Wojciech Ryś, Iaroslav Lutsyk, Karol Szałowski, Maxime Le Ster, Maciej Rogala, Michał Piskorski, Paweł Krukowski, Paweł Dąbrowski, Rafał Dunal, Aleksandra Nadolska, Przemysław Przybysz, Klaudia Toczek, Witold Kozłowski, Paweł J. Kowalczyk

**Affiliations:** 1 Department of Solid State Physics, Faculty of Physics and Applied Informatics, University of Lodz, Pomorska 149/153, 90-236 Łódź, Poland; 2 Zernike Institute for Advanced Materials, University of Groningen, Nijenborgh 4, 9747 AG Groningen, The Netherlands

**Keywords:** BiTeCl, Bi_2_Te_3_, Thermal degradation, Laser-induced degradation, Thermoelectric materials, Exfoliation energy, Materials chemistry, Condensed-matter physics, Thermoelectrics

## Abstract

This study examines the thermal stability and structural behavior of bismuth chloride telluride BiTeCl, a layered thermoelectric material with significant potential for energy conversion applications. Our investigations reveal that the chlorine-terminated surface exhibits poorer quality and increased defectivity compared to the tellurium-terminated side. The crystals were subjected to thermal annealing up to 520 K and green laser irradiation. Through techniques such as low-energy electron diffraction (LEED) and X-ray photoelectron spectroscopy (XPS), we found that while annealing enhances the crystallinity of the chlorine side up to 470 K, it ultimately suffers from thermal degradation above this temperature. The event leads to transformation into bismuth telluride - Bi_2_Te_3_ characterized by a lower BE shift of the Bi and Te bands by ~ 0.35 eV. The identity of the product was later confirmed via Raman spectroscopy while irradiating it with little laser power. With an increase in the power to 3.1 mW, it was however observed that the samples become locally modified undergoing similar degradation as during the annealing. The research demonstrates and characterizes the phenomena occurring during the decomposition either via irradiation or annealing. The explanation of such phenomena is then proposed based on the results of our theoretical DFT calculations. Additionally, we assess the usefulness of BiTeCl as a thermoelectric material, compare it in regard to the literature, and suggest new potential applications that may benefit from the transformation into Bi_2_Te_3_.

## Introduction

In the modern world, one of the most basic needs is energy generation for human civilization. The processes of production and usage of energy always lead to large thermal losses^1,2^. It is not surprising that the search for all kinds of green technologies and methods to reduce energy waste is an extremely important and popular topic^3,4^. One of the environmentally friendly ways to solve the problem of wasting energy on heat losses seems to be the use of thermoelectric materials – in which, as a result of the Seebeck effect, heating of such materials leads to the generation of electric potential^5–7^, thus efficiently, getting the most out of the produced electricity. Currently, many thermoelectric materials are known, mostly from the groups like chalcogenides, such as tellurides, selenides, or sulfides as well as semiconductive polymers, small-molecule organic compounds, halide perovskites, composite and topological materials^8^, although still major improvements in their efficiency and the development of new solutions are required. Efficient thermoelectric materials should possess a large Seebeck coefficient, high electrical conductivity, and low thermal conductivity^9^. One of the promising materials studied in this respect seems to be BiTeCl.

**Fig. 1:**
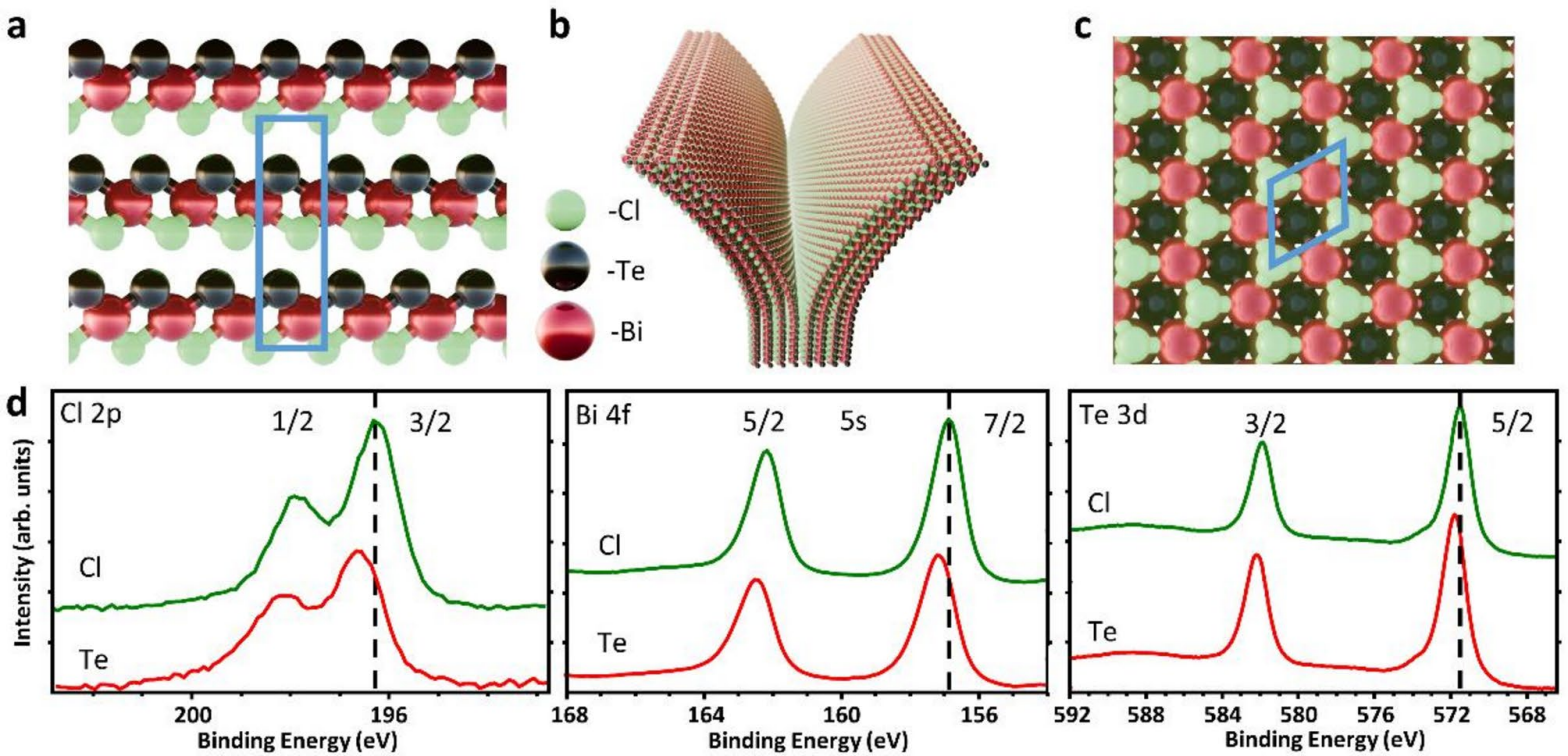
(**a**) Schematic side-view representation of the crystallographic structure of BiTeCl. The blue rectangle marks the unit cell. (**b**) A rendering illustrating schematically the process of exfoliating the material into two different sides (terminated by Cl or Te). (**c**) A top-view rendering of the crystallographic structure of BiTeCl. The blue rectangle marks the unit cell. d) XPS spectra collected for both sides of the vacuum-exfoliated BiTeCl crystal (red color indicates the side terminated with tellurium atoms, green—with chlorine atoms). Detailed spectra are presented respectively for the Cl 2p 3/2 & 2p 1/2, Bi 4f 7/2 & 4f 5/2, Bi 5s (negligible intensity) and Te 3d 5/2 & 3d 3/2, core lines.

Bismuth chloride telluride belongs to the group of bismuth tellurohalides – layered materials with the formula BiTeX, where X = F, Cl, Br, or I. Like BiTeBr, BiTeCl is a topological insulator^10,11^ as well as a Janus material, which for layered materials means that both sides of the crystal are terminated by different atoms or functional groups^6,11^. BiTeCl has a hexagonal crystal lattice with P63mc^7,12^ space group (see Fig. 1a and c). Consequently, it forms layers in which bismuth atoms are covalently bonded to chlorine and tellurium atoms^12^. On the other hand, neighboring layers are bound by intermolecular van der Waals forces. The termination of each side is homogeneous (purely Cl or Te terminated) and exhibits different properties depending on whether we examine the side terminated by the plane of chlorine or tellurium atoms^12–14^. This property results in each of the sides having a net electric charge of a different value. Bulk BiTeCl is a semiconductor with a bandgap value of approx. 0.7–0.8 eV^7,12^. However, due to the breaking of inversion symmetry, topological surface states emerge^10,11^. All the abovementioned features as well as the presence of properties such as the Rashba effect^12–16^, the high value of spin-orbit coupling originating from the presence of heavy atoms^6,17^, and the occurrence of the thermoelectric effect^6,7^, make it a potential candidate for applications in the fields such as optoelectronics^6^, spintronics^16,17^ or for example, as the beforementioned conversion of waste heat into electricity.

Despite the interesting properties and high application potential of BiTeCl described above, the thermal stability and possible structural changes in the material under the influence of elevated temperature have not yet been sufficiently investigated, which is of crucial importance for future electrothermal applications. It is known that materials can drastically change various properties as a result of laser irradiation^18–20^ and thermal annealing^21,22^. This gap in research hinders the development of reliable and efficient devices, as understanding the material’s behavior under operational temperatures is essential for ensuring long-term performance and durability. Comprehensive studies are needed to explore the thermal decomposition mechanisms and the material’s stability at elevated temperatures. Such investigations would provide critical insights into optimizing BiTeCl for thermoelectric and other high-temperature applications, paving the way for advancements in energy conversion technologies.

In our work, we investigate the thermal stability of the BiTeCl crystal in UHV conditions while annealing and in an inert atmosphere while subjected to laser irradiation and compare it with another work^23^. During the characterization of the opposite sides of BiTeCl, we observed worse quality and greater defectivity of the Cl terminated side than Te one. What is more, while annealing the crystal to improve the surface of the chlorine side, it was found that in practice its thermal stability range is not very broad, as is demonstrated by X-ray photoelectron spectroscopy (XPS), low-energy electron diffraction (LEED), and Raman spectroscopy. In the end, we try explaining the observed BiTeCl behavior via DFT calculations.

### Experimental methods

The examined samples were prepared from commercially purchased BiTeCl crystal. XPS and LEED measurements were carried out at room temperature in the UHV Scienta Omicron system. The base pressure in the chamber during the measurements was approx. 1 × 10^− 9^ mbar. As an X-ray source, a non-monochromatic DAR 400 with the Mg Kα line (1253.64 eV) source was used. The system is equipped with a hemispherical Phoibos 150 (SPECS) analyzer with a 2D-CCD detector, and the pass energy parameter was set to 30 eV. The XPS spectra were collected using the SpecsLab Prodigy software and processed with CasaXPS. The spectra were not shifted in any way, yet the observed C-C bond of carbon 1s has an energy of 284.8 eV. LEED (OCI Vacuum Microengineering Inc.) device was used to perform LEED measurements, and WSxM 5.0^24^ was used to process the results. The samples were subjected to annealing via resistive heating in the preparation chamber of the same UHV system, each time for 30 min. The sample was located above the heating element, which resulted in an upward heat transfer across its volume, up to the examined, top surface. The Raman spectrometer used (SOL Instruments) operates in a glove box under inert atmosphere conditions (argon)^25^. The used laser line was 532 nm, with a diffraction grating of 1800 gr/mm. Spectra were calibrated with respect to the residual signal of elastically scattered laser light. Raman maps were acquired on a 20 μm x 20 μm area, 40 by 40 points, 35s/point, and laser power of 0.2 mW. The black color corresponds to low signal intensity. The acquired Raman spectra were analyzed with Origin(Pro) 2021b. AFM measurements were carried out using an NT-MDT atomic force microscope using silicon TipsNano NSG01 tips in tapping mode. To process the AFM data, Gwyddion 2.59 software was used.

The theoretical calculations were performed using the Quantum ESPRESSO suite^26,27^, which implements density functional theory on a plane wave basis. We carried out calculations for bulk BiTeCl phase as well as monolayer and multilayers of thickness between 2 and 6 ML. For the latter case, a slab geometry was used, with at least 19 Å of vacuum separating the studied system from its periodic images. The lattice constants and atomic positions were fully relaxed, following the Broyden–Fletcher–Goldfarb–Shanno quasi-Newton algorithm. In calculations, we used scalar relativistic pseudopotentials (for lattice constant relaxations) and fully relativistic pseudopotentials^28^ (in other cases) with the projector augmented wave method^29^ and Perdew-Burke-Ernzerhof exchange-correlation functional^30^. We set the cut-off energies for charge density and wavefunctions to 402 Ry and 53 Ry, respectively. Van der Waals long-range interactions were accounted for in the semiempirical approach DFT-D2^31,32^. Moreover, a dipole correction was applied^33^ for slab geometries. The non-collinear calculations took into account spin-orbit coupling. The self-consistent calculation was performed on an 18 × 18 × 1 (18 × 18 × 6) grid of k-points for slabs (bulk) geometry. For relaxation, the convergence thresholds for total energy and total force were set to 10^− 6^ Ry and 10^− 4^ Ry/a_0_, respectively.

## Results and discussion

Firstly, the BiTeCl crystals were examined with XPS to investigate the differences between the Te- and Cl- terminated sides of the crystal. For this purpose, a single crystal was split into two pieces that were then exfoliated under UHV conditions (6 × 10^− 7^ mbar). A schematic view of different sides of the material after exfoliation is shown in the rendered image in Fig. 1b. The differences in chemical shifts shown in Fig. 1d are observed for the bismuth, tellurium, and chlorine bands between the opposite sides of the crystal. The resulting shifts are attributed to band bending^14,34^ and equal approx. 0.3–0.35 eV for all the orbitals, which makes them consistent with the data cited in the literature^13,14,34^. The 4f line of bismuth and 3d line of tellurium are shown as usually^35^ due to their higher multiplet separation and narrower full width at half maximum than for 5d bismuth and the 4d tellurium lines^13,14,34^.

**Fig. 2:**
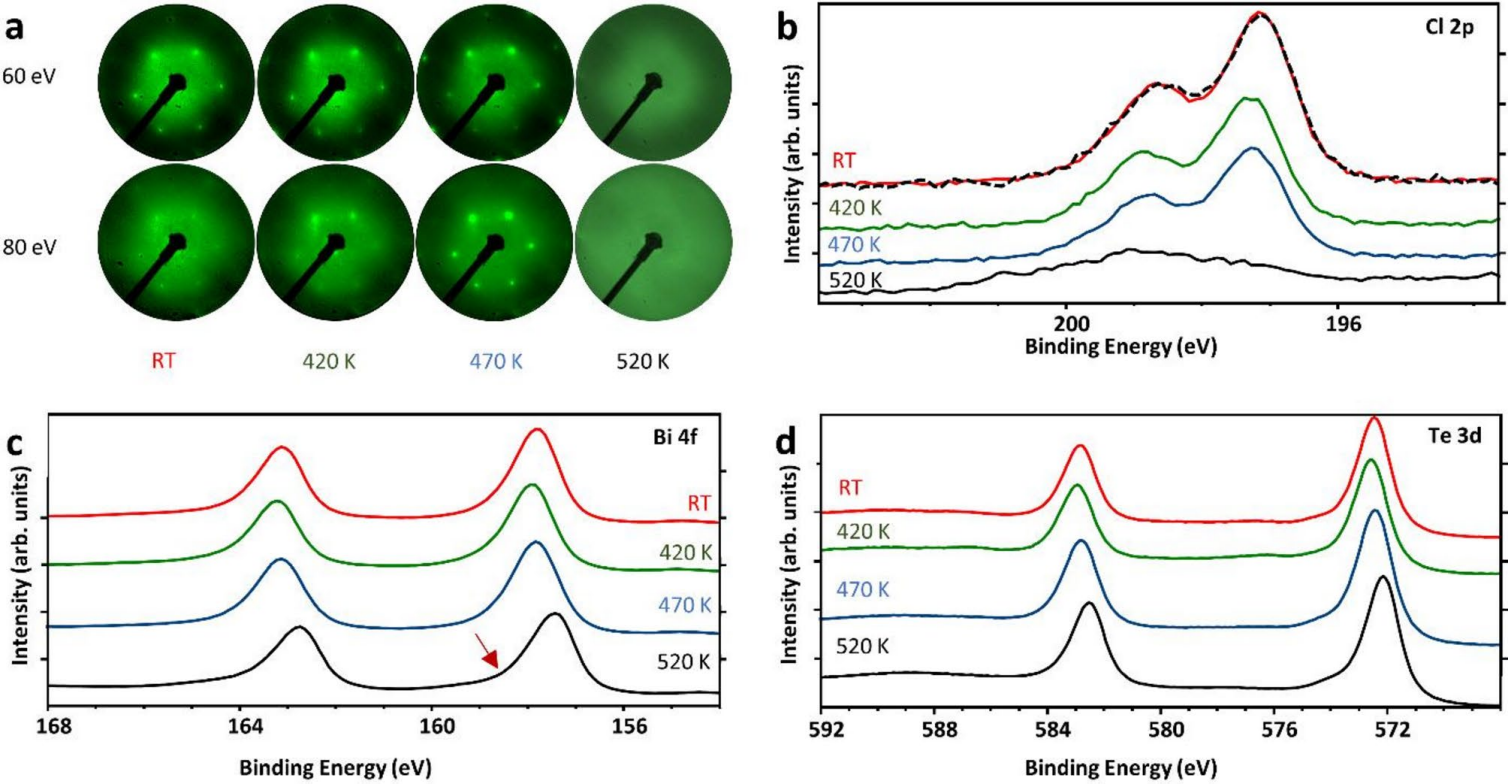
(**a**) LEED diffraction patterns obtained after different annealing temperatures (RT to 520 K, 60 eV, and 80 eV, respectively) for the chlorine-terminated side of the BiTeCl crystal. Respective XPS spectra for different annealing temperatures for core lines: (**b**) Cl 2p, (**c**) Bi 4f, (**d**) Te 3d. The black dotted curve in b) was measured for the post-annealed re-exfoliated crystal.

In order to get a deeper understanding of the surface of the exfoliated crystals, LEED and XPS measurements were carried out. By comparing LEED diffraction patterns obtained at room temperature for different electron energies (thus different elastic scattering conditions and possibly different surface penetration depth due to differing electron mean free paths^36^, based on the spots’ sharpness and intensity, it is possible to qualitatively infer the surface structure quality and the level of defectiveness. For the chlorine side, it was observed that the resulting pattern is less sharp and fainter than for the tellurium one (see Supplementary Fig. S1). In an attempt to improve the quality of the chlorine side of the crystal and induce reorganization of its surface, we decided to gradually anneal the sample. Changes caused by the annealing were studied using LEED and XPS spectroscopy (after the sample returned to thermal equilibrium). The diffraction images (see Fig. 2. a) indicate improvement in surface crystallinity as the annealing temperature increases. The sharpness of the diffraction patterns captured both at 60 eV and at 80 eV consistently showed a gradual improvement that peaked at 470 K. At the same time, a minor gradual loss of signal from chlorine atoms was observed on the XPS spectra acquired after heating at 470 K and below, it may be explained by a partial Cl desorption. The formation of hypothetical Cl vacancies in turn could lead to an increasing level of defectiveness. However, after annealing at 520 K there was a drastic change in the properties of the sample. The chlorine spectrum was changed significantly, and its intensity was greatly reduced (black curve 520 K, Fig. 2b). Simultaneously, the doublets corresponding to bismuth and tellurium exhibited a slight shift of approximately 0.35 eV toward lower binding energy (Fig. 2c,d). One possible explanation for this shift is that the dissociation of polarized chlorine–bismuth bonds results in the electron orbitals contracting back toward the Bi atom. This contraction increases the local negative charge density^13,37^, leading to the observed shift toward lower BE. However, this hypothesis does not fully account for why the magnitude of the energy shift is nearly identical for both Bi and Te atoms. As Te atoms are not directly involved in the dissociation of Cl bonds, their binding energy should be affected to a lesser extent. Regardless of the underlying mechanism, the measured Te 3d and Bi 4f peak positions remain within the typical binding energy range reported for Bi₂Te₃, consistent with previous literature^35,38,39^.

**Fig. 3:**
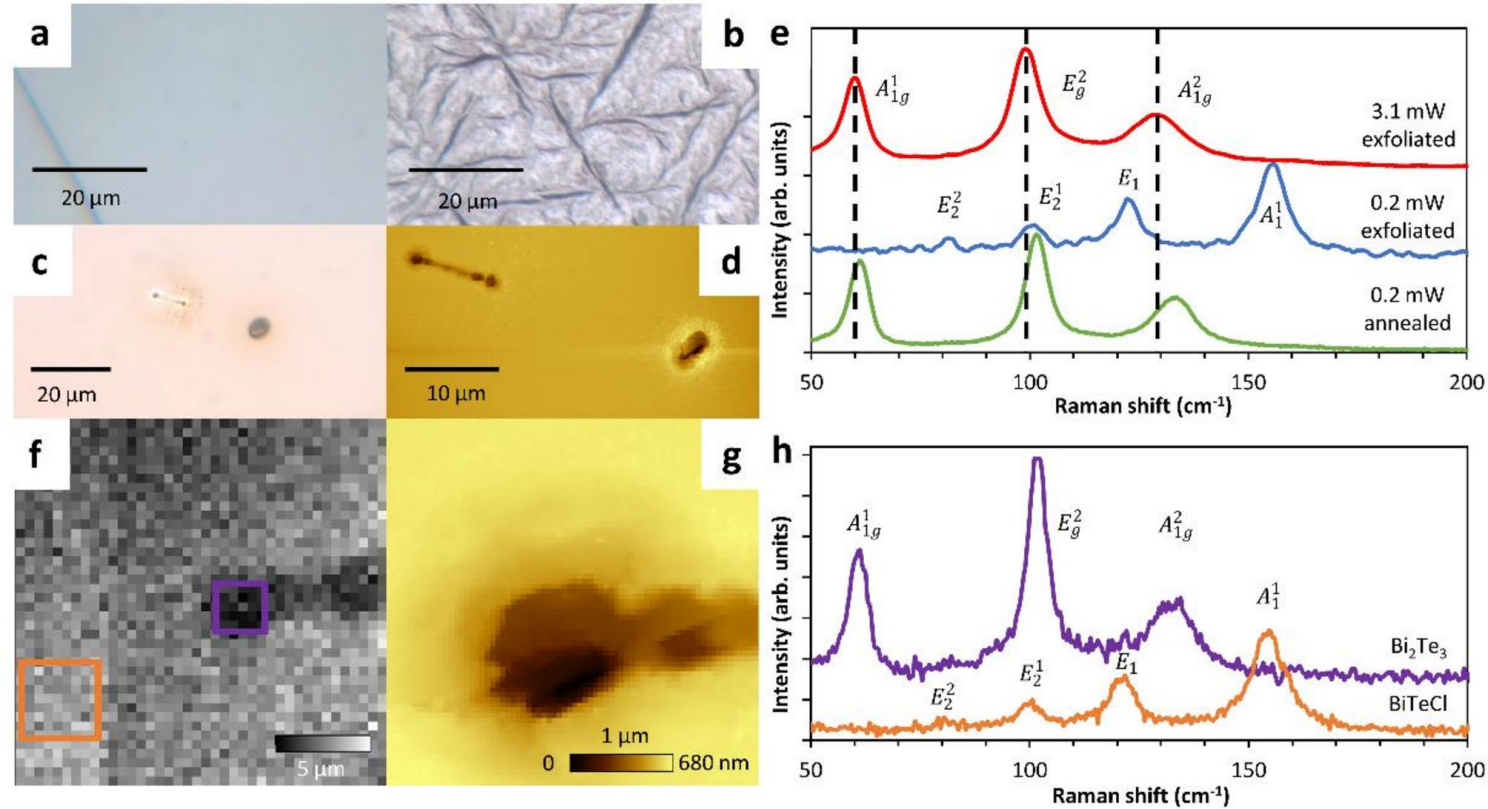
Optical images of: (**a**) freshly exfoliated BiTeCl crystal, (**b**) after thermal treatment at 520 K, (**c**) laser modification sites on the exfoliated BiTeCl surface. (**d**) Corresponding topography image obtained by AFM. (**e**) A set of Raman point spectra. The red spectrum - acquired at full laser power (3.1 mW) for a freshly exfoliated surface of a BiTeCl, which leads to a pointwise thermal modification. The blue spectrum - acquired with reduced power (0.2 mW) for an exfoliated surface. The green spectrum - acquired with reduced power (0.2 mW) for an annealed crystal. (**f**) Raman map corresponding to the $\:{A}_{1}^{1}$ BiTeCl peak captured at the crater site after a pointwise scan. (**g**) A corresponding AFM image. (**h**) The Raman spectra corresponding to the areas marked by orange and purple squares on the Raman map. In the first case, typical BiTeCl modes were identified on the spectrum, and in the second case typical for Bi_2_Te_3_.

Moreover, from the quantitative analysis, it was estimated that the ratio of Bi/Te changed from ~ 1 at RT to ~ 0.6 after the final annealing, in good accordance with the expected stichometry changes. In addition, a slight change of the bismuth bond to a more metallic one was observed – the peak shape has a larger shoulder^40^ on the high binding energy side of the bands (Fig. 2c marked with an arrow). Surprisingly, the diffraction pattern previously visible on LEED completely vanished. That suggests that the surface of the sample underwent severe changes that can be probably attributed to either the amorphization of the surface layers or the fact that it has undergone major macroscopic changes – from previously being metallic-silver, smooth, and reflective (Fig. 3a), after annealing it became matt-grey, rough, and cracked (Fig. 3b). Moreover, both of the above mentioned may hold true, and prevent the diffraction pattern from being obtained.

Due to the roughness, it was also impossible to examine the surface with AFM, as the height differences exceeded the order of hundreds of nanometers. However, this phenomenon did not alter the entire volume of the material, as the crystals, after further exfoliation, show a pure, unchanged surface of BiTeCl, the same as for a pre-annealed crystal (Fig. 3a). Consequently, all the XPS spectra after re-exfoliation look pretty much the same as the non-annealed sample. Even the main indicator of the change, the previously reduced Cl 2p band got restored to its former intensity and shape (see black dotted line on top of the red curve shown in Fig. 2b).

To gain a better understanding of the processes and changes that occur during the thermal modification of the samples, Raman spectroscopy measurements were conducted. For this purpose, a fresh, non-annealed piece of BiTeCl crystal was exfoliated in a glove box under inert atmosphere conditions and measured with the (commonly used by us) green laser (532 nm) with a power of 3.1 mW. Surprisingly, the measured Raman spectra obtained for samples prepared this way (red curve −3.1 mW exfoliated Fig. 3e) cannot be attributed to the characteristic bands of BiTeCl^41–43^. In addition, irradiation of the surface during measurements, even within seconds, resulted in the formation of holes with a depth of a few hundred nanometers (see optical and AFM images in Fig. 3c, d). These holes were usually surrounded by a few micrometers wide halo, thus resembling craters. The occurrence of these phenomena prompted us to carry out measurements with reduced laser power, in order to check whether they would be still invasive to the tested material.

Then, the measurements done with a power of 0.2 mW for a freshly exfoliated crystal led to Raman spectra that are typical of BiTeCl (see blue curve in Fig. 3e denoted 0.2 mW exfoliated). Four bands could be distinguished, i.e.: the weakest $\:{E}_{2}^{2}$ at 80 cm^− 1^, $\:{E}_{2}^{1}$ at 100 cm^− 1^, $\:{E}_{1}$ at 121 cm^− 1^ and $\:{A}_{1}^{1}$ at 154 cm^− 1^. These measurements unambiguously indicate the susceptibility of BiTeCl to change due to irradiation with a 532 nm green laser. It holds true even for a normally used operating power of 3.1 mW.

Having a reference in the form of a non-annealed crystal, we proceeded to collect spectra for the sample that was previously annealed to 520 K. The analysis of the curves collected both at full laser power (see supplementary Fig. S4) and with reduced power (green curve 0.2 mW annealed, Fig. 3e) consistently showed that they differ from those typical for BiTeCl. The acquired spectra were identified as characteristic of a topological insulator other than BiTeCl, that is bismuth telluride - Bi_2_Te_3_^44–46^. For that compound, we observe the following peaks: $A_{{1g}}^{1}$ at 59–61 cm^− 1^, $\:{E}_{g}^{2}$ at 98–102 cm^− 1^ and $A_{{1g}}^{2}$ at 128–133 cm^− 1^.

This information suggests the occurrence of similar processes for both the 520 K annealed sample and for freshly exfoliated crystal followed by 3.1 mW laser irradiation. For this reason, we claim that both processes; global macroscopic annealing and local laser irradiation, (probably as a result of the energy dissipation and very low thermal conductance^6,7^ local overheating occurs) can be associated with the same phenomenon of thermal degradation of the material. As an explanation, we propose the following thermolysis reaction, which is also a reversal of the usual BiTeCl synthesis reaction^7,10,43^:

1 $${\text{3BiTeCl}}~\mathop \to \limits^{{~T~}} {\text{Bi}}_{{\text{2}}} {\text{Te}}_{{\text{3}}} + {\text{BiCl}}_{{{\text{3}} \uparrow }}$$

Similar processes have been described and measured under other conditions using thermogravimetric analysis^23,47^. The abovementioned independently proposed reaction finds its confirmation in the work of Xin et al.^23^ and Marinković et al.^47^. In the former, the process of thermal decomposition of BiTeCl and BiTeBr was studied (with the main focus on the second compound), however, it was observed at a higher temperature of 600 K. In the latter work, it is hinted at by an analogous investigation of the BiTeI decomposition process. The results obtained in these works are generally consistent with our conclusions. However, our results show that under UHV conditions, due to the reduced pressure, the energy required for degradation is lower and the reaction can occur at temperatures lower than 600 K^23^ (approx. 520 K at 10^− 7^-10^− 8^ mbar). This temperature is also high enough for the formed bismuth chloride to be in a gaseous state or for its sublimation to occur – about 520 K^48^.

This is also confirmed by Raman mapping recorded in the area of the crater formed by the laser and in the adjacent region (Fig. 3f, g). The crater shows a strong Bi_2_Te_3_-like Raman spectrum, while the neighboring area is typical of BiTeCl (Fig. 3h, upper, purple, and lower, orange curves respectively). We hypothesize that gaseous bismuth chloride, formed during the laser-induced sublimation, leaves the system resulting in a build-up of pressure, and thus, causing the ejection of material around the hole, resulting in the formation of a crater (optical images and AFM Fig. 3c, d,g, additional ones in supplementary information Fig. S2, including 3D projections of the Fig. 3g in Fig. S3). Such holes are created in less than seconds (at 3.1 mW), and the resulting indentations are over hundreds of nanometers (potentially even thousands of nm) deep at the deepest points, which makes it impossible to thoroughly examine such places with AFM. The resulting holes have a spherical shape resembling that of a laser beam and a size of fractions to even a few µm depending on the exposure time. Around the edge of the cavity, a small, spherical, halo-like material elevation is observed, probably caused by either gas ejection and/or thermal decomposition of impurities on the surface. The presence of BiCl_3_ or its potential derivatives formed by contact with water/moisture, i.e. the intermediate hydrate BiCl_3_ ∙ H_2_O or BiOCl, could not be detected on the sample either by Raman spectroscopy or XPS. Despite the occurrence of Bi_2_Te_3_ characteristic modes $A_{{1g}}^{1} ,E_{g}^{2}$ and $A_{{1g}}^{2}$ both after sample annealing and laser irradiation, in these respective cases, they are shifted relatively to each other by several (between 1.0 and 4.5) inverse centimeters (see Fig. 3e). This shift is unequal for each of the modes. As it was found out by comparing measurements taken with different power, with and without annealing (see supplementary Fig. S3), it is the laser-induced temperature increase that is responsible for the blueshift of the band. The way Bi_2_Te_3_ was obtained seems insignificant in this regard. Besides the blueshift, all the peaks at elevated temperatures experience broadening. The FWHM values increase on average by approx. 1 cm^− 1^, 3 cm^− 1^, and 10 cm^− 1^, for $A_{{1g}}^{1} ,E_{g}^{2}$ and $A_{{1g}}^{2}$ peaks respectively. The observed peak broadening and frequency decrease with the increasing temperature are expected phenomena related to temperature influence on phonon modes^49,50^.

**Fig. 4:**
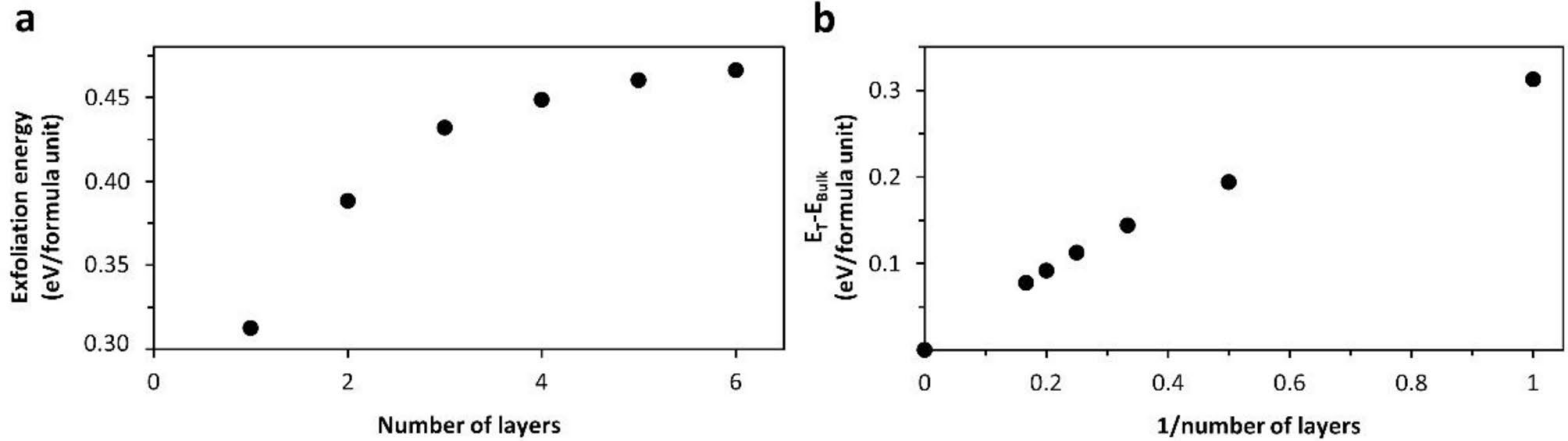
DFT calculations for BiTeCl. (**a**) Exfoliation energy versus number of exfoliated layers relation (up to 6 layers). (**b**) Differential total energy per formula unit versus 1/number of layers relation for bulk crystal and 1–6 layers. Total energy values are set relatively to the bulk by subtracting its energy value.

Our experiments have shown that the degradation of samples is associated with the decomposition of the near-surface area. To understand this phenomenon, we decided to undertake a density functional theory (DFT) study. We approached this in two steps. Firstly, we decided to compare the exfoliation energies for 1–6 layers from the bulk crystal. As seen in Fig. 4a, a monolayer (ML) exfoliation is highly preferential and most probable. Thus fewer the layers the easier it gets to remove them from bulk. As for the second step, we calculated the total energy per layer for different numbers of layers (1–6) and a bulk material. These calculations indicate that the energy per layer is 0.31 eV higher for the ML than for the bulk, and 0.19 eV higher for 2 L than for the bulk (Fig. 4b). With each additional layer approaching bulk, the energy value per layer decreases, indicating the increasing stability of the material with thickness. Combining these two steps we propose a hypothesis justifying why according to our XPS and Raman results, in the tested temperature range, the material does not change in its volume, but only in a thin layer near the surface. The relatively low energy required to remove the ML (or a thin layer) from the bulk makes it delaminate whilst heating. Then while not supported by the rest of the material, the less stable ML undergoes a thermal decomposition reaction, as the thinner the material the more prone to degradation it is due to the larger total energy (see Fig. 4b). From the Raman spectroscopy, it may be indicated that the process does not terminate in the first few, most outer layers as the material degradation advanced so deeply that we cannot see any traces of BiTeCl (Fig. 3e green curve 0.2 mW annealed). Thus we hypothesize that in UHV the process is not limited by any other factor but time. Should it be annealed long enough, it would yield a complete transformation into Bi_2_Te_3_. The calculations therefore coincide with the observed experimental results, potentially explaining why a degradation of the top layers of the material is preferred and why they are less stable with respect to the inside of the crystal.

Based on our work here, we suggest that the use of BiTeCl as a thermoelectric material may not be as effective as it is thought. For example, the reported theoretical thermal conductivity properties and the dimensionless figure of merit (ZT) of BiTeCl calculated for temperatures higher than approx. 520–600 K are unreliable and do not translate into the possible use of this material, either in the form of a bulk or as a monolayer^6^. Based on the reported levels of its efficiency, the narrow range of thermal stability of BiTeCl (> 520 K) limits its potential practical applications in comparison to, for example, the most popular thermoelectric material Bi_2_Te_3_ which is more effective. Moreover, the theoretical ab initio molecular dynamics calculations^51^, which predict the stability of BiTeCl at 600 K, obviously disagree with our experimental results. It raises our concern whether the limited range of operating temperature would allow to applicate BiTeCl as a thermoelectric material, still, it may work just fine for some lower temperature applications. Clearly, there is undergoing discussion regarding the usability of BiTeCl and more experiments are required to fully understand the potential of this van der Waals material in either bulk, thin layers, or hybrid forms.

### Summary and outlook

We characterized both sides of the BiTeCl crystal using LEED and XPS, finding the chlorine-terminated surface to be of lower quality. Consequently, we analyzed its stability under annealing. The Cl-terminated side proved unstable at elevated temperatures, as indicated by the reduction of the chlorine 2p signal. However, crystallinity improved up to 470 K, beyond which thermal degradation set in, leading to rapid surface (only) transformation into bismuth telluride. Additionally, laser irradiation induced analogical localized thermolysis, forming deep craters likely due to gas ejection. Pre-annealed samples showed no further changes under laser exposure, suggesting complete surface transformation to Bi₂Te₃. Theoretical calculations suggest that low energy barriers make thin BiTeCl layers prone to exfoliation and thermolysis, progressing from the surface inward while leaving the bulk intact. Given these findings, BiTeCl use in thermoelectric devices may be limited by the range of its operating temperatures, though it remains viable at lower temperatures. The observed laser-induced transformation could have potential applications in lithographic fabrication of small-scale heterojunctions or heterostructures that combine the properties of both materials or as a protective capping layer, improving system stability.

## Electronic supplementary material

Below is the link to the electronic supplementary material.

Supplementary Material 1

## Data Availability

The data are included in the article/supplementary material/referenced in the article. Raw data may also be requested from the corresponding authors.
