# Supplementary material for: Degradation of BiTeCl induced by thermal and laser treatment
Source: Sci Rep. 2025 May 7;15:15936. doi: 10.1038/s41598-025-00907-5 (PMC12059073; doi:10.1038/s41598-025-00907-5)
Supplement: Supplementary file 1 — Supplementary Material 1 [file 41598_2025_907_MOESM1_ESM.pdf]

Supplementary information for the manuscript "Degradation of BiTeCl induced by thermal and laser treatment"

Authors

Wojciech Ryś[a]*, Iaroslav Lutsyk[a], Karol Szałowski[a], Maxime Le Ster[a], Maciej Rogala[a], Michał Piskorski[a], Paweł Krukowski[a], Paweł Dąbrowski[a], Rafał Dunal[a], Aleksandra Nadolska[a], Przemysław Przybysz[a,b], Klaudia Toczek[a], Witold Kozłowski[a], Paweł J. Kowalczyk[a]*

[a] *Department of Solid State Physics, Faculty of Physics and Applied Informatics, University of Lodz, Pomorska 149/153, 90-236 Łódź, Poland.*
[b] *Zernike Institute for Advanced Materials, University of Groningen, Nijenborgh 4, 9747 AG Groningen, The Netherlands*
*Corresponding authors: wojciech.rys@edu.uni.lodz.pl (W. Ryś), pawel.kowalczyk@uni.lodz.pl (P.J. Kowalczyk)

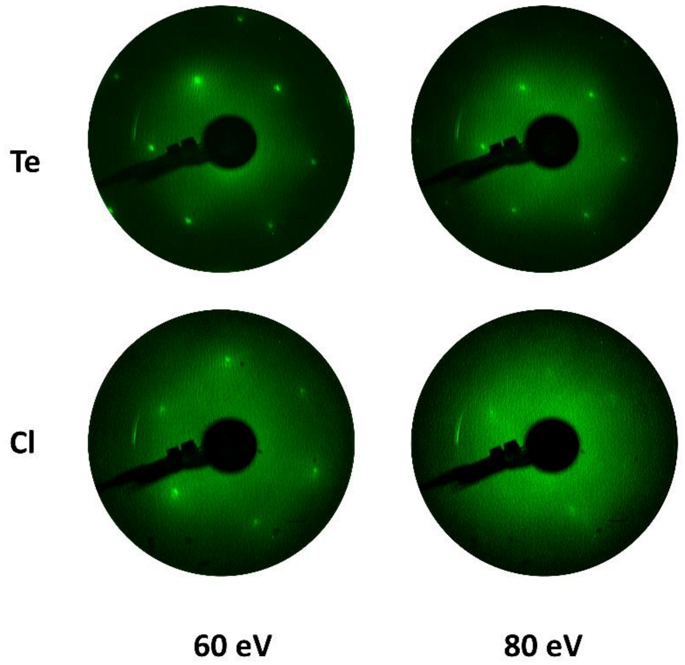

Fig. S1. LEED pattern images of the Te and the Cl terminations of the BiTeCl measured with beam energy equal to 60 and 80 eV.

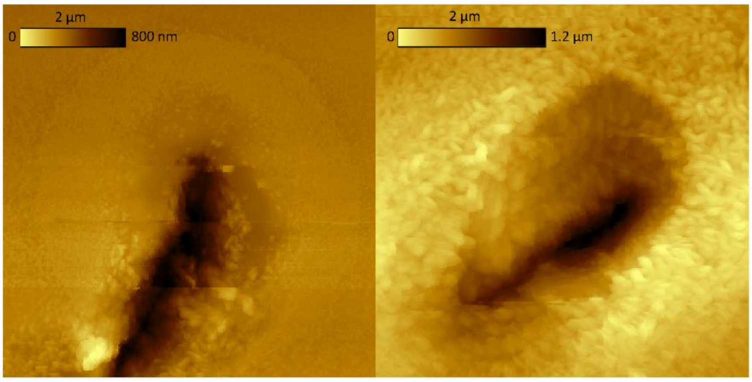

Fig. S2. AFM topographical images of two different crater sites created as a result of point irradiation.

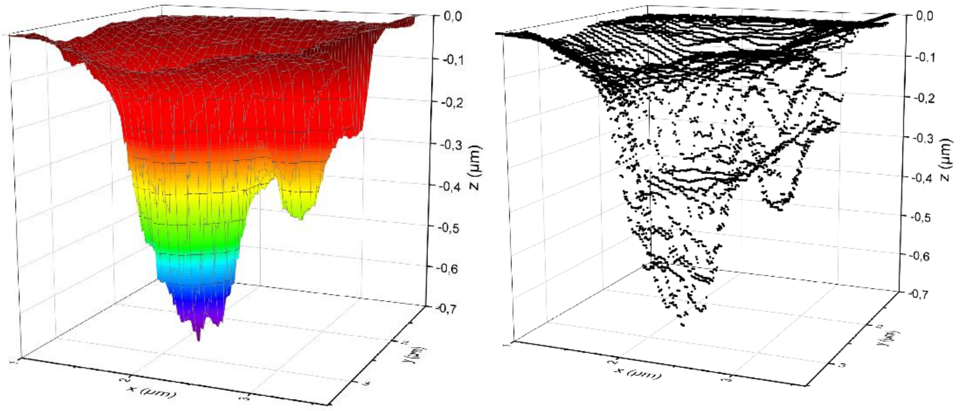

Fig. S3. The 3D representations of AFM topography from Fig. 3g.

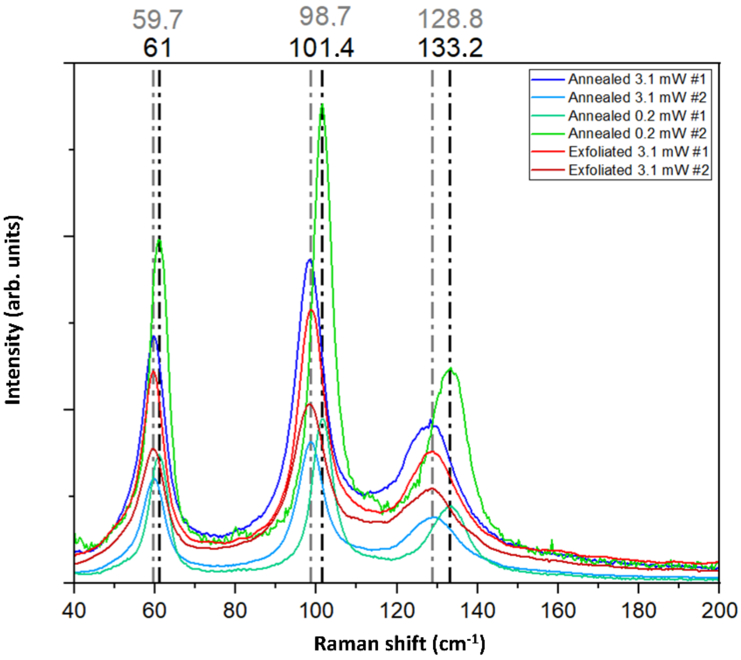

Fig. S4. Bi_2Te_3 Raman shift and annealing power relation. Measurements conducted with higher power lead to a temperature-induced blueshift of the bands.
